# Intranasal administration of *Lactobacillus johnsonii* attenuates hyperoxia-induced lung injury by modulating gut microbiota in neonatal mice

**DOI:** 10.1186/s12929-023-00958-8

**Published:** 2023-07-31

**Authors:** Chung-Ming Chen, Yu-Chen S. H. Yang, Hsiu-Chu Chou, Shan Lin

**Affiliations:** 1 Department of Pediatrics, Taipei Medical University Hospital, Taipei, Taiwan; 2 Department of Pediatrics, School of Medicine, College of Medicine, Taipei Medical University, Taipei, Taiwan; 3 TMU Research Center for Digestive Medicine, Taipei Medical University, Taipei, Taiwan; 4 Joint Biobank, Office of Human Research, Taipei Medical University, Taipei, Taiwan; 5 Department of Anatomy and Cell Biology, School of Medicine, College of Medicine, Taipei Medical University, Taipei, Taiwan; 6 Biotech Research Institute, Grape King Bio Ltd., Taoyuan, Taiwan

**Keywords:** Probiotics, Hyperoxia, Mean linear intercept, Vascular endothelial growth factor, Microbiota

## Abstract

**Background:**

Supplemental oxygen impairs lung development in newborn infants with respiratory distress. *Lactobacillus johnsonii* supplementation attenuates respiratory viral infection in mice and exhibits anti-inflammatory effects. This study investigated the protective effects of intranasal administration of *L. johnsonii* on lung development in hyperoxia-exposed neonatal mice.

**Methods:**

Neonatal C57BL/6N mice were reared in either room air (RA) or hyperoxia condition (85% O_2_). From postnatal days 0 to 6, they were administered intranasal 10 μL *L. johnsonii* at a dose of 1 × 10^5^ colony-forming units. Control mice received an equal volume of normal saline (NS). We evaluated the following four study groups: RA + NS, RA + probiotic, O_2_ + NS, and O_2_ + probiotic. On postnatal day 7, lung and intestinal microbiota were sampled from the left lung and lower gastrointestinal tract, respectively. The right lung of each mouse was harvested for Western blot, cytokine, and histology analyses.

**Results:**

The O_2_ + NS group exhibited significantly lower body weight and vascular density and significantly higher mean linear intercept (MLI) and lung cytokine levels compared with the RA + NS and RA + probiotic groups. At the genus level of the gut microbiota, the O_2_ + NS group exhibited significantly higher *Staphylococcus* and *Enterobacter* abundance and significantly lower *Lactobacillus* abundance compared with the RA + NS and RA + probiotic groups. Intranasal *L. johnsonii* treatment increased the vascular density, decreased the MLI and cytokine levels, and restored the gut microbiota in hyperoxia-exposed neonatal mice.

**Conclusions:**

Intranasal administration of *L. johnsonii* protects against hyperoxia-induced lung injury and modulates the gut microbiota.

**Supplementary Information:**

The online version contains supplementary material available at 10.1186/s12929-023-00958-8.

## Introduction

Respiratory distress syndrome is the most common cause of breathing difficulties in preterm newborns, affecting approximately 50% of all preterm infants born before 30 weeks of gestation [1]. Supplemental oxygen is frequently used to treat neonates who have respiratory problems. However, oxygen treatment for newborns can have adverse consequences. Prolonged hyperoxia exposure in newborn rats reduces alveolar septation and angiogenesis, increases the terminal air gap size, and impairs lung development [2, 3]. Bronchopulmonary dysplasia (BPD) is a chronic lung disease that develops in premature infants due to the damage caused by hyperoxia-induced lung injury and insufficient repair mechanisms in the developing lung [4]. BPD in neonates is not only attributed to postnatal risk factors such as oxidative stress and mechanical ventilation but also to respiratory and intestinal microbial dysbiosis [3, 5–7]. Microbial dysbiosis is linked to severe BPD [8]; the respiratory tract quickly becomes colonized after birth through exposure to both the mother and the environment. Independent studies have supported the role of the respiratory microbiome in the pathogenesis of BPD and have suggested that modifying the airway microbiome may reduce the risk of BPD [9]. Additionally, intestinal bacteria can affect lung tissue, and alterations in the gut microbiota may lead to a more severe BPD phenotype [6]. The interaction between the gut and lung is known as the gut–lung axis [10]. The relationship between alterations in intestinal and lung microbiota and BPD warrants further investigation.

Studies have demonstrated that various probiotics exhibit considerable antibacterial effects by modulating the immune system [11]. *Lactobacillus* spp. has been found to be effective in preventing or treating several respiratory diseases [12]. Although the oral administration of *Lactobacillus* spp. can be beneficial for preventing influenza infection in mice, bacteria may not successfully reach the gut intact when passing through the acidic conditions of the stomach [13]. Intranasal administration of probiotics increases the cellular immune response in adult mice and can prevent or treat respiratory virus or bacterial infection as a result of the direct augmentation of immune system [14–17]. Intranasal administration of recombinant *Lactococcus lactis* expressing heme oxygenase-1 reduces hyperoxia-induced lung inflammation in 21-day-old rats [18]. However, the protective effects of intranasal administration of *Lactobacillus* spp. on the hyperoxia-induced arrest of lung development in newborn animals are currently unknown. In the present study, we hypothesized that intranasal administration of *L. johnsonii* improves lung injury induced by hyperoxia by modulating the lung and intestinal microbiota in neonatal mice. Therefore, we evaluated the protective effects and underlying mechanisms of intranasal administration of *L. johnsonii* on hyperoxia-induced lung injury in neonatal mice.

## Materials and methods

### Experimental animals

Time-dated pregnant C57BL/6 mice were housed in individual cages with open access to laboratory food and water. Mice were kept under a 12-h light/12-h dark cycle and allowed to deliver vaginally at term. Within 12 h of birth, the litters were pooled, randomly redistributed to the newly delivered mothers, and then exposed to either hyperoxia (85% O_2_) or room air (RA) for 1 week. The commercial probiotic, *L. johnsonii* (strain GKJ2), was provided by Grape King Bio, Ltd. (Taoyuan, Taiwan). The blend was chosen based on the findings that *L. johnsonii* supplementation can attenuate respiratory viral infection in mice [19]. Before intranasal administration, the purified powders were freshly rehydrated immediately in normal saline (NS) at 10^7^ colony-forming units (CFU) per mL. The day of birth was considered postnatal day 0. A total of 10 μL *L. johnsonii* at 1 × 10^5^ CFU per dose was administered intranasally to each newborn mouse from postnatal days 0 to 6. We chose a dose of 10^5^ CFU because a higher dose of 10^6^ CFU resulted in diarrhea in neonatal mice. Control mice received an equal volume of NS. We divided the mice into the following four study groups: RA + NS, RA + probiotic, O_2_ + NS, and O_2_ + probiotic. The mice were exposed to oxygen in a modified controller (NexBiOxy, Hsinchu, Taiwan) for maintaining precise oxygen concentrations. The nursing mothers were rotated between the O_2_ treatment and RA control litters every 24 h to avoid oxygen toxicity and eliminate the maternal effects between the treatment subgroups. The oxygen-rich atmosphere was maintained in a transparent 40-cm × 50-cm × 60-cm plexiglass chamber that received O_2_ continuously at 4 L/min. The oxygen concentration inside the plexiglass hyperoxic chamber was continually monitored using an oxygen sensor (Coy Laboratory Products, Grass Lake, MI, USA).

### Mouse tissue collection and processing

Survival rates were recorded daily. Animals were euthanized with an overdose of isoflurane, and the blood was drawn using a sterile needle through the right ventricle. The lung and lower gastrointestinal tract were harvested on postnatal day 7. We sampled the microbiota from the lower gastrointestinal tract by using a culture-independent approach (i.e., community sequencing of the 16S rRNA gene on the Illumina MiSeq System). To ensure sterility, instruments were washed with ethanol and flamed after each tissue harvest. The murine lungs were excised, placed in tubes containing 1 mL of sterile water, and homogenized mechanically by using a Tissue-Tearor (Biospec Products, Bartlesville, OK, USA). The tissue homogenizer was cleaned and rinsed in ethanol and water after each tissue harvest.

### 16S rRNA gene sequencing

16S rDNA analysis was conducted according to the methods of Yang et al. [20]. 16S rDNA was purified from stool by using the QIAamp Fast DNA Stool Mini Kit (QIAGEN, Hilden, Germany) and purified from lung tissue by using the QIAamp DNA Microbiome Kit (QIAGEN). Library preparation was conducted following the protocol of the 16S Ribosomal RNA Gene Amplicons for the Illumina MiSeq System. Sequence reads were submitted to the European Nucleotide Archive under the accession number PRJEB28574. In accordance with the protocol, the gene‐specific sequences targeting the 16S V3 and V4 region were removed from the demultiplexed paired reads by using Cutadapt (v. 1.12). By following the workflow described by Callahan et al. [21], the R package DADA2 (v. 1.14.1) was used to process the filtered reads in the R environment (v. 3.6.1). Taxonomic assignments were performed using the SILVA database (v. 128) [22] as a reference, with a minimum bootstrap confidence of 80. Multiple sequence alignment of the amplicon sequence variants (ASVs) was performed using DECIPHER (v. 2.14.0), and the phylogenetic tree was constructed through alignment by using the phangorn package (v. 2.5.5) [23]. The count table, taxonomic assignment results, and phylogenetic tree were merged into a phyloseq object, which was then used for community analyses through the phyloseq package (v. 1.30.0) [24]. The α-diversity index was calculated using the estimate_richness function of the phyloseq package. Statistical comparisons between the treatment and control groups were performed using the Kruskal–Wallis and Wilcoxon tests, and the significance level (i.e., α) was set at 0.05. The community dissimilarity between the groups was assessed by calculating UniFrac distances using the GUniFrac package (v. 1.1) [25]. Principal coordinate analysis (PCoA) ordination on the UniFrac distances was also performed. The vegan package (v. 2.5.6) was used to determine dissimilarities in group compositions and the homogeneity of dispersion. Specifically, the adonis and betadisper functions were used to perform a statistical analysis of these measures. To analyze enrichment between the groups, the linear discriminant analysis (LDA) Effect Size (LEfSe) method was used, with the Wilcoxon–Mann–Whitney test set at a significance level of α = 0.05 and a logarithmic LDA score of greater than 3 [26]. The results were visualized as a cladogram by using GraPhlAn [27]. Lung microbial metabolic function was predicted by Phylogenetic Investigation of Communities by Reconstruction of Unobserved States (PICRUSt2) based on the 16 s rRNA gene quantification and sequencing results [28]. ALDEx2 was run on each pair of samples separately and used to determine significantly increased and decreased metabolic activities (effect size > 0.5).

### Western blot of lung vascular endothelial growth factor

Lung tissues were homogenized in ice-cold buffer containing 50 mM Tris·HCl (pH 7.5), 1 mM EGTA, 1 mM EDTA, and a protease inhibitor cocktail (complete minitablets; Roche, Mannheim, Germany). The samples were sonicated and then centrifuged at 500×*g* for 20 min at 4 °C to remove cellular debris. Proteins (30 μg) were resolved through sodium dodecyl sulfate–polyacrylamide gel electrophoresis (SDS-PAGE) on 12% SDS-PAGE gel under reducing conditions and electroblotted to a polyvinylidene difluoride membrane (Immobilon-P, Millipore, Bedford, MA, USA). After the membranes were blocked with 5% nonfat dry milk, they were incubated with antibodies against vascular endothelial growth factor (VEGF) (1:750; SC-152, Santa Cruz Biotechnology, Dallas, TX, USA) and anti-β-actin (1:1000; Sigma-Aldrich, St. Louis, MO, USA), and they were subsequently incubated with horseradish peroxidase–conjugated goat anti-rabbit or mouse immunoglobulin G (IgG) (Pierce Biotechnology, Rockford, IL, USA). Protein bands were detected using SuperSignal Substrate (Pierce Biotechnology). A densitometric analysis was performed on the AIDA program to measure the intensity of the VEGF and β-actin bands.

### Western blot of intestine ZO-1 and occludin

The ileum was homogenized, sonicated, and centrifuged at 12,000×*g* for 20 min at 4 °C to remove cellular debris. Proteins (30 μg) were resolved on 8% SDS-PAGE gel under reducing conditions and electroblotted onto polyvinylidene difluoride membranes (Immobilon-P, Millipore). After the membranes were blocked with 5% nonfat dry milk, they were incubated with anti-occludin (1:1000; ThermoFisher Scientific, Waltham, MA, USA) and anti-ZO-1 antibodies (1:250; Abcam) or anti-β-actin (1:1000; Santa Cruz Biotechnology), and they were then incubated with horseradish peroxidase–conjugated goat anti-rabbit or anti-mouse IgG antibodies (GeneTex, San Antonio, TX, USA). Protein bands were detected using the SuperSignal Substrate (Pierce Biotechnology). Densitometric analysis was performed to measure the intensity of the occludin, ZO-1, and β-actin bands. Data were normalized to β-actin for each mouse.

### Lung cytokine levels

Approximately 100 mg of lung tissue from each pup was homogenized using Procarta lysis buffer (Affymetrix, Santa Clara, CA, USA) according to the manufacturer’s instructions. The cell extracts were centrifuged, and the levels of IL-1, IL-6, and TNF-α in the supernatants were determined using the Bio-Plex multiplex assay system (Bio-Rad, Hercules, CA, USA).

### Lung morphology

To standardize the analysis, tissue sections were taken from the middle lobe of the right lung. We stained 5-μm sections of lung tissue with hematoxylin and eosin, examined them using light microscopy, and assessed them for lung morphometry and fibrosis. The mean linear intercept (MLI), an indicator of the mean alveolar diameter, was assessed in 10 nonoverlapping fields [29]. We objectively determined the vascular density by analyzing a minimum of five random lung fields stained with the von Willebrand factor (vWF). The results are expressed as a vWF-positive vessel per high power field [30].

### Immunohistochemical staining for von Willebrand factor

Immunohistochemical staining was performed on 5-μm paraffin sections. After the paraffin sections were deparaffinized, heat-induced epitope retrieval was performed by immersing the sections in 0.01 M sodium citrate buffer (pH 6.0). To block endogenous peroxidase activity and the nonspecific binding of antibodies, the sections were preincubated for 1 h at room temperature in 0.1 M phosphate-buffered saline containing 10% normal goat serum and 0.3% H_2_O_2_. The sections were incubated with rabbit polyclonal anti-vWF antibodies (1:200, GTX60934; GeneTex, Irvine, CA, USA) for 20 h at 4 °C and biotinylated goat anti-rabbit IgG (1:200, Jackson ImmunoResesarch Laboratories, West Grove, PA, USA) for 1 h at 37 °C. The avidin–biotin complex kit (Vector Laboratories, Newark, CA, USA) and diaminobenzidine substrate kit (Vector Laboratories) were used to visualize the brown reaction product according to the manufacturer’s recommendations. All immunostained sections were viewed and photographed using an Olympus BX43 microscope. Digital images of each section were captured and quantified by counting the positive stained vessels in five randomly selected fields per section at × 400 magnification.

### Statistical analysis

All data are presented as mean ± SD. Statistical analyses were performed using two-way analysis of variance (ANOVA) and a Bonferroni post hoc test for multiple group comparisons. The survival rate was calculated using the Kaplan–Meier method, and a log-rank test was used to compare groups. The α-diversity index was evaluated using Kruskal–Wallis with one-way ANOVA and a Bonferroni post hoc test. The development parameters and the correlations between the gut bacteria at the genus level (i.e., *Staphylococcus*, *Enterobacter*, *and Lactobacillus*) and lung injury were analyzed using Spearman’s rank correlation test. The differences were considered statistically significant at *p* < 0.05.

## Results

### Survival rate

Five female mice delivered 37 pups. Seven to eleven pups were randomly redistributed among the new mothers after the litter was pooled. The mice reared in hyperoxia exhibited respiratory distress. All mice housed in RA or hyperoxia and treated with NS or the probiotic survived on postnatal day 7.

### Intranasal *L. johnsonii* administration increased hyperoxia-induced decrease in body weight

**Fig. 1:**
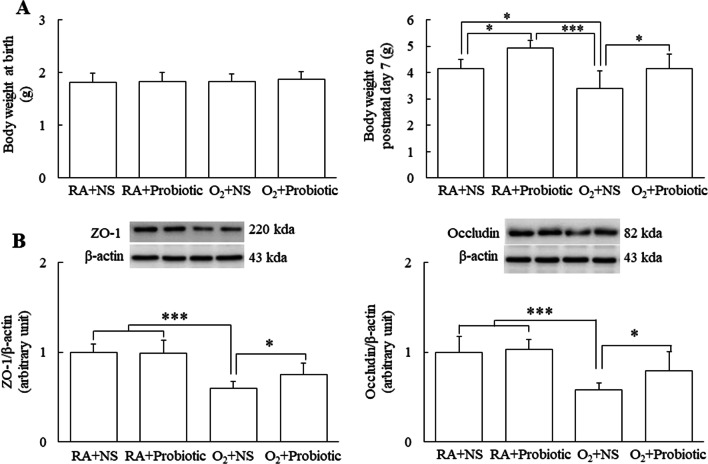
Intranasal *L. johnsonii* administration increased the body weights and intestinal junctional protein expression in hyperoxia-exposed neonatal mice on postnasal day 7. Intranasal *L. johnsonii* or NS was administered to neonatal mice reared in RA or O_2_ from postnatal days 0–6. **A** Birth body weights and body weights on postnatal day 7, and **B** representative Western blots and semiquantitative analysis of intestinal ZO-1 and occludin expression on postnatal day 7. *n* = 7–11. *p < 0.05, ***p < 0.001, two-way ANOVA followed by Bonferroni post hoc test

The mean birth weights were similar among the four study groups (Fig. 1A). The mice reared in RA and treated with *L. johnsonii* exhibited significantly greater body weight than those reared in RA and treated with NS. The mice reared in the hyperoxia condition and treated with *L. johnsonii* exhibited significantly lower body weight than those reared in RA and treated with NS or *L. johnsonii*. Treatment with *L. johnsonii* significantly increased the hyperoxia-induced decrease in body weight on postnatal day 7.

### Intranasal *L. johnsonii* administration increased intestinal junctional protein expression

Epithelial tight junctions are formed by the transmembrane proteins ZO-1 and occludin, which control intercellular permeability and are vital for the integrity of the intestinal barrier. We measured ZO-1 and occludin protein expression to evaluate the probiotic effect on intestinal barrier integrity. The mice reared in the hyperoxia condition and treated with NS exhibited significantly lower ZO-1 and occludin protein expression than those reared in RA and treated with NS or *L. johnsonii* (Fig. 1B). Treatment with *L. johnsonii* significantly increased the hyperoxia-induced decrease in ZO-1 and occludin protein expression compared with treatment with NS.

### Intranasal *L. johnsonii* administration improved alveolarization and increased VEGF expression and lung angiogenesis

**Fig. 2:**
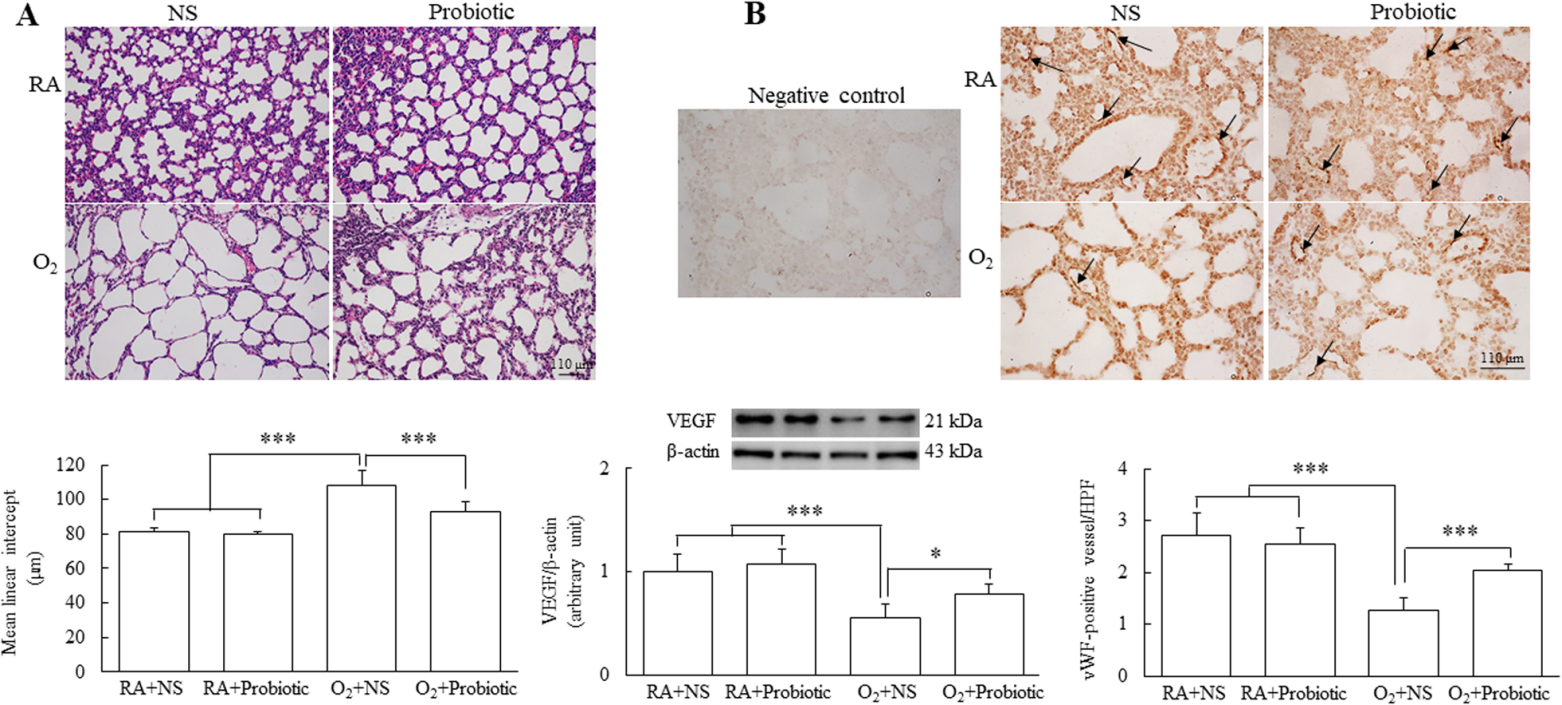
Intranasal *L. johnsonii* administration improved alveolarization and increased VEGF expression and lung angiogenesis on postnatal day 7. **A** Representative lung histology and MLI on postnatal day 7. **B** Representative immunohistochemical staining for vWF (black arrow) and representative Western blots and semiquantitative analysis of VEGF and vascular density. Treatment with *L. johnsonii* significantly diminished the hyperoxia-induced increase in the MLI and significantly augmented the hyperoxia-induced decrease in VEGF protein levels and vascular density. *n* = 7–11. *p < 0.05, ***p < 0.001, two-way ANOVA followed by Bonferroni post hoc test

On postnatal day 7, lung tissue sections stained with hematoxylin and eosin were examined (Fig. 2A). The mice reared in RA and treated with NS exhibited normal lung morphology. The mice reared in the hyperoxia condition and treated with NS exhibited large thin-walled air spaces and a significantly higher MLI level than those reared in RA and treated with NS or *L. johnsonii*. Treatment with *L. johnsonii* significantly diminished the increase in MLI level induced by hyperoxia. Figure 2B presents the representative immunohistochemistry staining of vWF in lung tissue sections. VEGF is a powerful endothelial cell mitogen that is required for angiogenesis. The mice reared in the hyperoxia condition and treated with NS exhibited significantly lower vWF immunoreactivity. A semiquantitative analysis revealed that the mice reared in the hyperoxia condition and treated with NS exhibited significantly lower VEGF expression and vascular density than those reared in RA and treated with NS or *L. johnsonii* (Fig. 2B). Treatment with *L. johnsonii* significantly augmented the decrease in VEGF protein levels and vascular density induced by hyperoxia more than treatment with NS.

### Intranasal *L. johnsonii* administration decreased the hyperoxia-induced increase in lung cytokines

**Fig. 3:**
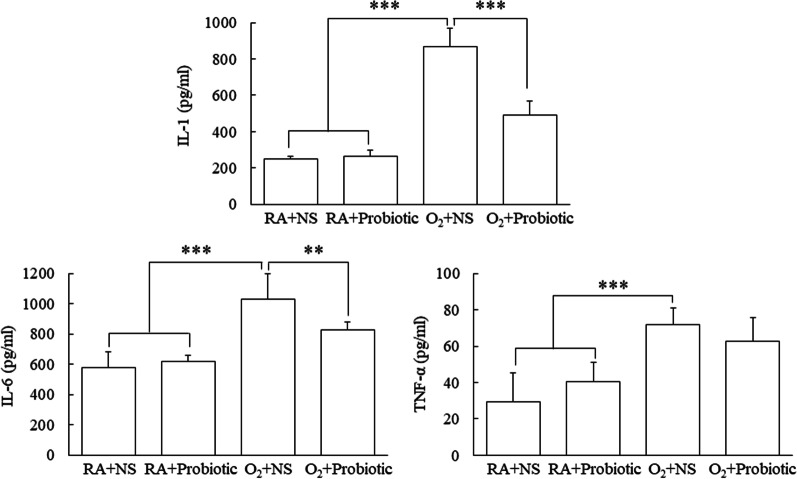
Intranasal *L. johnsonii* administration decreased the hyperoxia-induced increase in lung cytokines. The mice reared in hyperoxia and treated with NS exhibited significantly higher lung IL-1, IL-6, and TNF-α levels compared with those reared in RA and treated with NS or *L. johnsonii*. Treatment with *L. johnsonii* significantly inhibited the hyperoxia-induced increase in lung IL-1 and IL-6 levels on postnatal day 7.* n* = 7–11. **p < 0.01, ***p < 0.001, two-way ANOVA followed by Bonferroni post hoc test

The mice reared in the hyperoxia condition and treated with NS exhibited significantly higher lung IL-1, IL-6, and TNF-α levels than those reared in RA and treated with NS or *L. johnsonii* (Fig. 3). Treatment with *L. johnsonii* significantly inhibited the increase in lung IL-1 and IL-6 levels induced by hyperoxia on postnatal day 7.

### Intranasal *L. johnsonii* administration modulated the gut microbiota

**Fig. 4:**
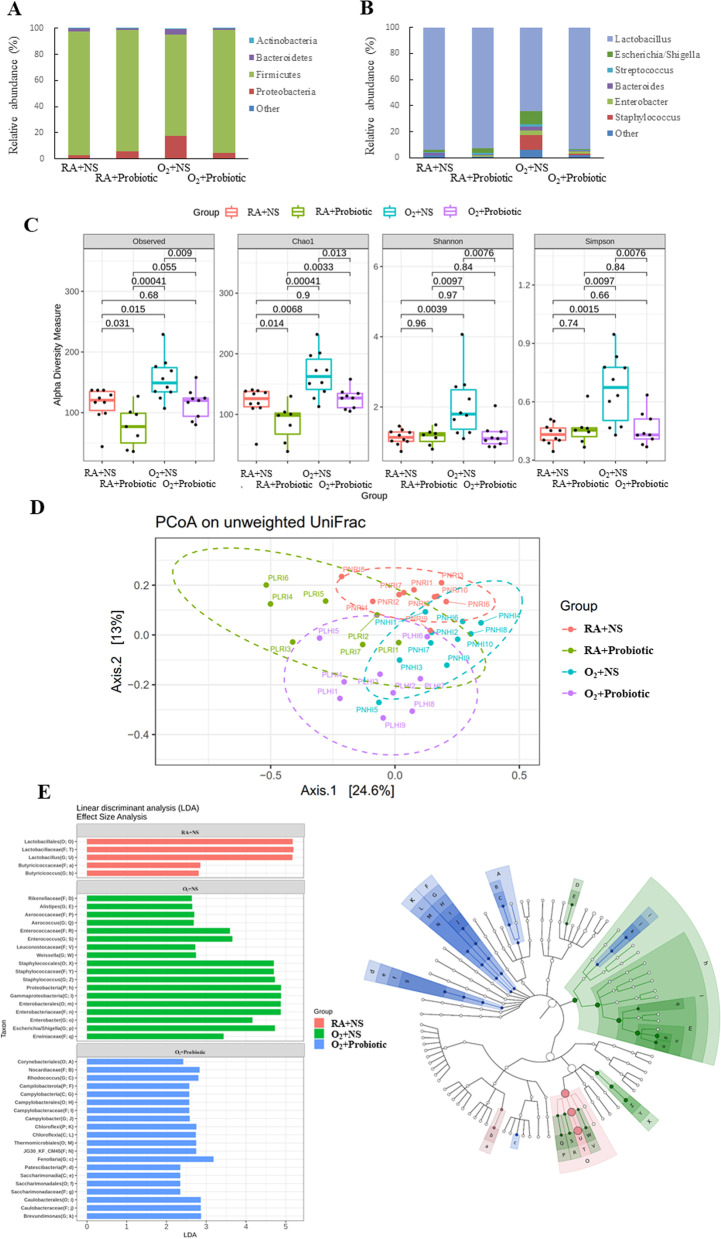
Intranasal *L. johnsonii* administration–modulated gut microbiota in hyperoxia-exposed neonatal mice. **A** Bacterial composition at the phylum level. **B** Bacterial composition at the genus level. **C** α-diversity. **D** β-diversity. **E** Linear discriminant analysis. Intranasal *L. johnsonii* administration altered the bacterial composition and diversity of the gut microbiota in the O_2_ + NS (*n* = 10), RA + NS (*n* = 7), RA + probiotic (*n* = 10), and O_2_ + probiotic (*n* = 9) groups. Two-way ANOVA followed by Bonferroni post hoc test. PNR: RA + NS, PLR: RA + probiotic, PNH: O_2_ + NS, PLH: O_2_ + probiotic

The microbiota compositions were analyzed at the phylum and genus levels to explore the microbial species and their relative abundance. At the phylum level, the first four phyla (i.e., *Firmicutes*, *Proteobacteria*, *Bacteroidetes*, and *Actinobacteria*) accounted for more than 99% of the sequences in the four groups (Fig. 4A). The gut microbiota mainly consisted of *Proteobacteria* and *Bacteroidetes* in the O_2_ + NS group, and *L. johnsonii* treatment nonsignificantly decreased *Proteobacteria* and *Bacteroidetes* abundance. At the genus level, the first six genera (*Lactobacillus*, *Staphylococcus*, *Enterobacter*, *Bacteroides*, *Streptococcus*, and *Escherichia/Shigella*) accounted for more than 97% of the sequences in the four groups (Fig. 4B). The O_2_ + NS group exhibited significantly higher *Staphylococcus* (*p* < 0.01) and *Enterobacter* (*p* < 0.05) abundance and significantly lower *Lactobacillus* abundance (*p* < 0.01) than the RA + NS and RA + probiotic groups. Administration of *L. johnsonii* significantly decreased the abundance of the genera *Staphylococcus* and *Enterobacter* and increased the abundance of *Lactobacillus*.

As shown in Fig. 4C, the gut microbiome of the O_2_ + NS group exhibited significantly higher α-diversity than the RA + NS and RA + probiotic groups. *L. johnsonii* administration significantly decreased the α-diversity of the O_2_ group, and the α-diversity values approached those of the RA + NS and RA + probiotic groups. Additionally, the PCoA on unweighted UniFrac distances revealed a significant difference between these groups (Fig. 4D, p < 0.001). We conducted an LDA to identify the differences in microbiome compositions between the groups. As shown in Fig. 4E, *Lactobacillales* (order), *Lactobacillaceae* (family), *Lactobacillus* (genus), *Butyricicoccaceae* (family), and *Butyricicoccus* (genus) decreased after O_2_ or *L. johnsonii* administration. *Rikenellaceae* (family), *Alistipes* (genus), *Aerococcaceae* (family), *Aerococcus* (genus), *Enterococcaceae* (family), *Enterococcus* (genus), *Leuconostocaceae* (family), *Weissella* (genus), *Staphylococcales* (class), *Staphylococcaceae* (order), *Staphylococcus* (family), *Proteobacteria* (phylum), *Gammaproteobacteria* (class), *Enterobacterales* (order), *Enterobacteriaceae* (family), *Enterobacter* (genus), *Escherichia_Shigella* (genus), and *Erwiniaceae* (genus) abundance was high in the O_2_ + NS group. *Corynebacteriales* (order), *Nocardiaceae* (family), *Rhodococcus* (genus), *Campilobacterota* (phylum), *Campylobacteria* (class), *Campylobacterales* (order), *Campylobacteraceae* (family), *Campylobacter* (genus), *Chloroflexi* (phylum), *Chloroflexia* (class), *Thermomicrobiales* (order), *JG30_KF_CM45* (family), *Fenollaria* (genus), *Patescibacteria* (phylum), *Saccharimonadia* (class), *Saccharimonadales* (order), *Saccharimonadaceae* (family), *Caulobacterales* (order), *Caulobacteraceae* (family), and *Brevundimonas* (genus) abundance was high in the O_2_ + probiotic group.

### Effects of intranasal *L. johnsonii* administration on lung microbiota

**Fig. 5:**
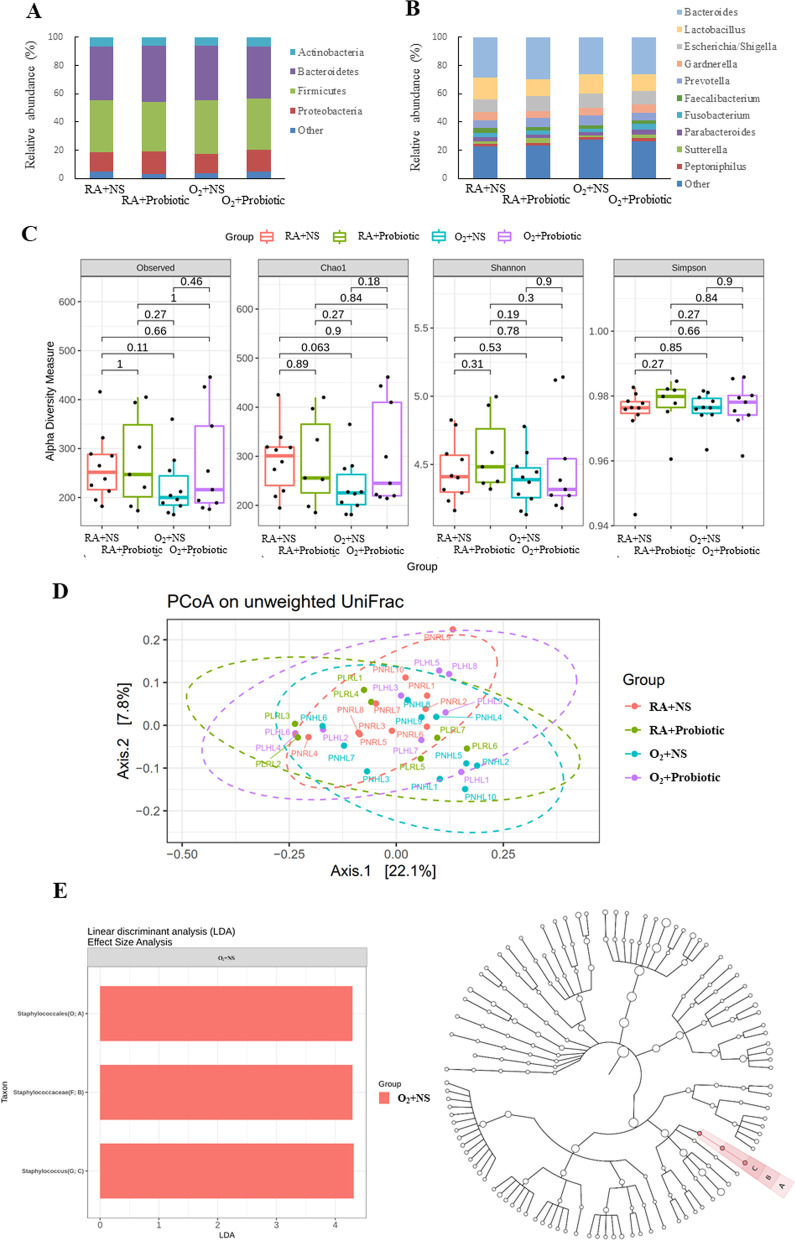
Effects of intranasal *L. johnsonii* administration on the lung microbiota.** A** Bacterial composition at the phylum level. **B** Bacterial composition at the genus level. **C** α-diversity. **D** β-diversity. **E** Linear discriminant analysis. Intranasal *L. johnsonii* administration altered the bacterial composition and diversity of the lung microbiota in the O_2_ + NS (*n* = 10), RA + NS (*n* = 7), RA + probiotic (*n* = 10), and O_2_ + probiotic (*n* = 9) groups. Two-way ANOVA followed by Bonferroni post hoc test. PNR: RA + NS, PLR: RA + probiotic, PNH: O_2_ + NS, PLH: O_2_ + probiotic

At the phylum level, the first four phyla (i.e., *Firmicutes*, *Bacteroidetes*, *Proteobacteria*, and *Actinobacteria*) accounted for > 95% of the sequences in the four groups (Fig. 5A). At the genus level, the first 10 genera accounted for > 75% of the sequences in the four groups (Fig. 5B). The microbiota compositions were comparable among the four groups at both the phylum and genus levels. In the richness analysis of the lung microbiota, α-diversity was not significantly different among the four groups (Fig. 5C). The PCoA on unweighted UniFrac distances revealed a significant difference between the groups (Fig. 5D, p < 0.05). As shown in Fig. 5E, *Staphylococcales* (order), *Staphylococcaceae* (family), and *Staphylococcus* (genus) abundance was high in the O_2_ + NS group.

### Correlation between dominant gut microbiota and lung injury parameters

**Fig. 6:**
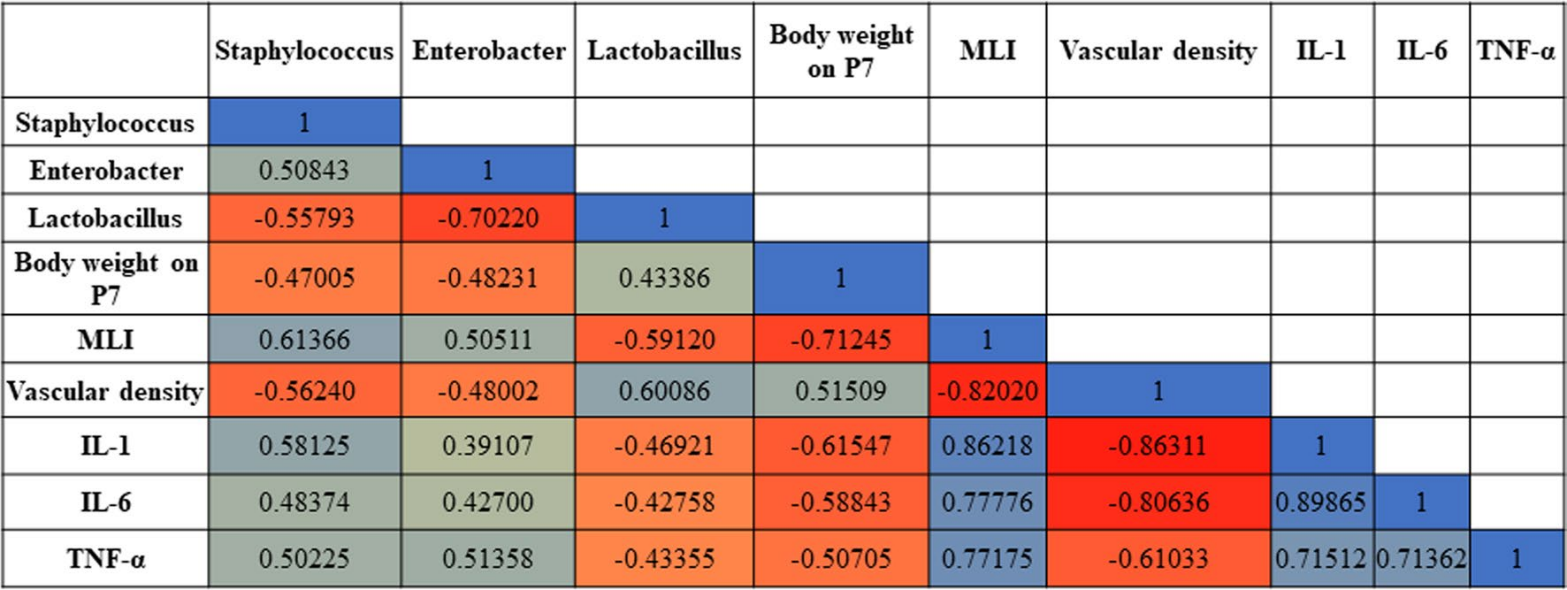
Correlation heatmap of the dominant gut microbial genera, lung development, and lung injury parameters. The blue color indicates a positive correlation. The red color indicates a negative correlation

To determine which specific bacteria attenuated hyperoxia-induced lung injury, a Spearman correlation was performed to compare the dominant bacteria at the genus level with the lung development parameters and inflammatory markers (Fig. 6). The abundance of *Staphylococcus* and *Enterobacter* was positively correlated with both the level of MLI and lung cytokines and negatively correlated with body weight on postnatal day 7 and vascular density. However, the abundance of *Lactobacillus* was negatively correlated with the level of MLI and lung cytokines and positively correlated with body weight on postnatal day 7 and vascular density.

### Effects of *L. johnsonii* on predicted metabolic pathways

**Fig. 7:**
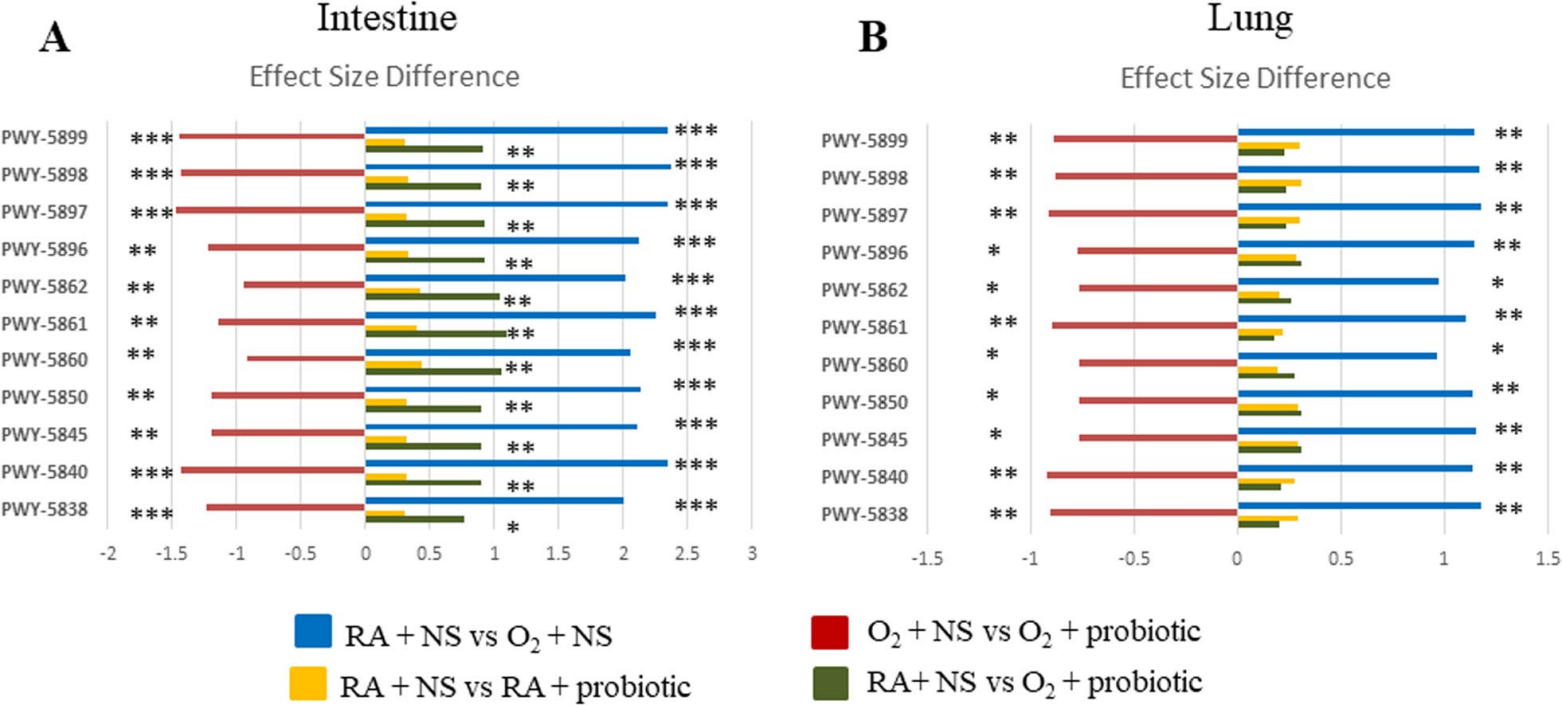
Vitamin K2-related biosynthetic pathways of differentially abundant predicted metabolic pathways in the comparison between hyperoxia or *L. johnsonii* treatment (**A**) in intestine and (**B**) in lung. Abundance analysis was performed using ALDEx2 (*p < 0.05; **p < 0.01; ***p < 0.001)

The metabolic pathways of the intestine and lungs were predicted based on microbial communities of four groups (Additional file 1, Additional file 2, Additional file 3, Additional file 4, Additional file 5, Additional file 6, Additional file 7, Additional file 8). In the results, among the metabolic pathways induced by hyperoxia treatment, there was a significant difference in the biosynthetic pathways of vitamin K2. Therefore, the biosynthetic pathways related to vitamin K2, including PWY-5838, PWY-5840, PWY-5845, PWY-5850, PWY-5860, PWY-5861, PWY-5862, PWY-5896, PWY-5897, PWY-5898, PWY-5899 (Table 1), were selected and discussed. Hyperoxia treatment increased the metabolic activities of predicted metabolic pathways of vitamin K2 biosynthesis including PWY-5838, PWY-5840, PWY-5845, PWY-5850, PWY-5860, PWY-5861, PWY-5862, PWY-5896, PWY-5897, PWY-5898 and PWY-5899 (Fig. 7A and B). Treatment with *L. johnsonii* decreased metabolic activities of vitamin K2 biosynthesis, and the effect of PWY-5838, PWY-5840, PWY-5845, PWY-5850, PWY-5861, PWY-5896, PWY-5897, PWY-5898 and PWY-5899 was lower than -0.5 (Fig. 7A and B). In addition, only RA + NS and O_2_ + probiotic groups showed significance with effects greater than 0.5 in the intestine (Fig. 7A), but not in the lung (Fig. 7B). The biosynthetic pathway of vitamin K2 was not significantly affected between RA + NS and RA + probiotic groups (Fig. 7A and B). These results indicate that hyperoxia treatment-induced vitamin K2 biosynthesis pathways were reduced by *L. johnsonii* treatment in the intestine and lung.

**Table 1:** Description of the pathways

Pa thway	Description
PWY-5838	Superpathway of menaquinol-8 biosynthesis I
PWY-5840	Superpathway of menaquinol-7 biosynthesis
PWY-5845	Superpathway of menaquinol-9 biosynthesis
PWY-5850	Superpathway of menaquinol-6 biosynthesis I
PWY-5860	Superpathway of demethylmenaquinol-6 biosynthesis I
PWY-5861	Superpathway of demethylmenaquinol-8 biosynthesis
PWY-5862	Superpathway of demethylmenaquinol-9 biosynthesis
PWY-5896	Superpathway of menaquinol-10 biosynthesis
PWY-5897	Superpathway of menaquinol-11 biosynthesis
PWY-5898	Superpathway of menaquinol-12 biosynthesis
PWY-5899	Superpathway of menaquinol-13 biosynthesis

## Discussion

Our animal model revealed that exposure to high oxygen concentration during the first 7 days following delivery can lead to lower body weight, higher lung cytokine levels, and impaired lung development in neonatal mice. Intranasal administration of *L. johnsonii* improved lung development, which was demonstrated by lower MLI and cytokine levels and higher VEGF expression and vascular density. The improvement in lung development was accompanied by the normalization of the intestinal microbiota. We found that intranasal administration of *L. johnsonii* may suppress lung inflammation and protect against hyperoxia-induced lung injury. Thus, intranasal administration of *L. johnsonii* is a potential treatment for hyperoxia-induced lung injury in newborns.

In this study, all the mice had similar body weights at birth and received similar nutrients in their food. We found that the mice reared in RA or the hyperoxia condition and treated with *L. johnsonii* exhibited a significantly higher body weight than those treated with NS. The mice reared in the hyperoxia condition and treated with *L. johnsonii* exhibited significantly higher protein expression in the intestinal junction than the mice reared in RA and treated with NS. These results suggest that probiotics protect the integrity of the intestinal mucosa and promote digestion and absorption. These findings are compatible with those of Li et al., who found that mice supplemented with probiotics exhibited higher activity of intestinal digestive enzymes and had a higher body weight without increasing their food intake [31].

Soluble factors from *L. reuteri* significantly reduced the production of proinflammatory cytokines (i.e., IL-6 and TNF-α) by lipopolysaccharide-stimulated macrophages and alleviated lipopolysaccharide-induced acute lung injury in mice [32]. In a previous study, oral *L. johnsonii* administration decreased the expression of serum C-reactive protein and liver TNF-α mRNA and improved the gut environment in obese mice [33]. Our study found that *L. johnsonii* inhibited increases in lung cytokine levels induced by hyperoxia, which is consistent with the results of other studies; this finding indicated that *Lactobacillus* possesses anti-inflammatory effects.

By using the LEfSe algorithm, we identified specific taxa that were enriched or depleted in the microbiota of each group. Hyperoxia exposure during the first 7 days of life altered the bacterial community composition of the intestinal microbiota in the mice. Among the first six intestinal microbiota genera (i.e., *Lactobacillus, Staphylococcus, Enterobacter, Bacteroides, Streptococcus*, and *Escherichia/Shigella*), the abundance of *Staphylococcus* and *Enterobacter* significantly increased, and that of *Lactobacillus* significantly decreased in the O_2_ + NS group compared with the RA + NS and RA + probiotic groups. These results are consistent with those of Li et al., who indicated that hyperoxia promotes the growth of *Staphylococcus* spp. and *Enterobacter* spp. and inhibits the growth of *Lactobacillus* spp. [34]. *Lactobacillus* spp*.* was inhibited because of direct oxygen toxicity in anaerobic cells and the production of toxic oxygen by-products and reactive oxygen species, which may damage DNA, proteins, and lipids and result in cellular death [35].

Our previous study found that hyperoxia exposure from postnatal days 0 to 7 decreases tight junction proteins (occludin and ZO-1) expression, disrupts the intestinal barrier, increases intestinal permeability and bacterial translocation, and impairs intestinal function in newborn rats [36]. In the present study, we demonstrated that hyperoxia-exposed neonatal mice exhibited alterations in their intestinal microbiota, presented the increased abundance of *Staphylococcus* and *Enterobacter*, and demonstrated the reduction of *Lactobacillus* abundance. These changes in the intestinal microbial communities were associated with lower levels of proteins in the intestinal junction and improvements to hyperoxia-induced lung injury (i.e., decreased lung inflammation and improved lung alveolarization and angiogenesis). Wang et al. found that respiratory influenza in mice leads to changes in the intestinal microbiota. Moreover, they found that alterations in the microbial communities were not due to active influenza infection in the intestine, but finally resulted in gut immunological damage and inflammation [37]. Furthermore, intranasal administration of *L. johnsonii* prevented the gut microbiota disruption observed in hyperoxia-exposed neonatal mice, implying a link between impaired lung development and gut microbiota composition and supporting the existence of the gut-lung axis upon hyperoxia-induced lung injury.

*Lactobacillus* has a positive effect on the treatment of respiratory diseases, and a number of animal experiments and clinical trials have confirmed their efficacy [12]. A possible mechanism by which *Lactobacillus* affect the lung microenvironment through the gut-lung axis is via regulating the immune system through peptidoglycans, extracellular polysaccharides, surface proteins, and metabolites (short-chain fatty acids), and inorganic polyphosphate liquids. Another possible mechanism is that *Lactobacillus* affects the lung microenvironment through pattern recognition receptors (such as Toll-like receptors or NOD-like receptors, etc.) on immune cells in the respiratory mucosa and activate downstream pathways [12].

Our study has several limitations. First, we did not examine the association between the metabolites of intestinal microbial communities and immune homeostasis, although several potential links between such metabolites and immune homeostasis have been proposed to explain the gut–lung axis. Microbes or microbial components may be transported to the lung through lymphatic transport, whereas metabolites created or altered by the gut microbiota can affect lung physiology [38, 39]. Second, we did not analyze the nasal microbiota in neonatal mice. Although the nasal microbiome can predict BPD in preterm infants [40], the relationship between nasal microbiota and hyperoxia-induced lung injury remains unknown.

## Conclusion

In the present study, intranasal administration of *L. johnsonii* improved lung development in hyperoxia-exposed neonatal mice; decreased the MLI and lung cytokine levels; and increased the vascular density. The results also demonstrated that intranasal *L. johnsonii* administration increased neonatal body weight. Moreover, *L. johnsonii* could restore the intestinal microbiota upon hyperoxia-induced lung injury. No scientifically established therapy for improving lung damage caused by hyperoxia currently exists. However, intranasal *L. johnsonii* treatment may prevent hyperoxia-induced lung injury. Further research on the outcomes of controlling particular gut microbiota, such as supplementing the microbiota with *L. johnsonii*, may provide therapeutic alternatives for managing hyperoxia-related lung injury in neonates.

## Supplementary Information

**Additional file 1:** Excel file containing ALDEx2 output comparing intestinal metabolic pathways between RA + NS and RA + probiotic groups.

**Additional file 2:** Excel file containing ALDEx2 output comparing intestinal metabolic pathways between RA + NS and O_2_ + NS groups.

**Additional file 3:** Excel file containing ALDEx2 output comparing intestinal metabolic pathways between RA + NS and O_2_ + probiotic groups.

**Additional file 4:** Excel file containing ALDEx2 output comparing intestinal metabolic pathways between O_2_ + NS and O_2_ + probiotic groups.

**Additional file 5:** Excel file containing ALDEx2 output comparing lung metabolic pathways between RA + NS and RA + probiotic groups.

**Additional file 6:** Excel file containing ALDEx2 output comparing lung metabolic pathways between RA + NS and O_2_ + NS groups.

**Additional file 7:** Excel file containing ALDEx2 output comparing lung metabolic pathways between RA + NS and O_2_ + probiotic groups.

**Additional file 8:** Excel file containing ALDEx2 output comparing lung metabolic pathways between O_2_ + NS and O_2_ + probiotic groups.

## Data Availability

Data are available upon request. Most results from this study are included in the article.
